# Added value of systematic biopsy in men with a clinical suspicion of prostate cancer undergoing biparametric MRI-targeted biopsy: multi-institutional external validation study

**DOI:** 10.1007/s00345-020-03393-8

**Published:** 2020-08-10

**Authors:** Ugo Falagario, Ivan Jambor, Pekka Taimen, Kari T. Syvänen, Esa Kähkönen, Harri Merisaari, Ileana Montoya Perez, Juha Knaapila, Aida Steiner, Janne Verho, Ashutosh Tewari, Hannu J. Aronen, Giuseppe Carrieri, Peter J. Boström, Otto Ettala

**Affiliations:** 1 Department of Urology and Organ Transplantation, University of Foggia, Foggia, Italy; 2 Department of Urology, Icahn School of Medicine At Mount Sinai, New York, NY USA; 3 Department of Radiology, University of Turku, Turku, Finland; 4 Department of Radiology, Icahn School of Medicine At Mount Sinai, New York, NY USA; 5 Medical Imaging Centre of Southwest Finland, Turku University Hospital, Turku, Finland; 6 Institute of Biomedicine, University of Turku and Department of Pathology, Turku University Hospital, Turku, Finland; 7 Department of Urology, University of Turku and Turku University Hospital, Turku, Finland; 8 Department of Future Technologies, University of Turku, Turku, Finland

**Keywords:** Prostate cancer, PSA, MRI, Biparametric MRI, Targeted biopsy, Systematic biopsy

## Abstract

**Purpose:**

We aimed to develop and externally validate a nomogram based on MRI volumetric parameters and clinical information for deciding when SBx should be performed in addition to TBx in man with suspicious prostate MRI.

**Materials and methods:**

Retrospective analyses of single (IMPROD, NCT01864135) and multi-institution (MULTI-IMPROD, NCT02241122) clinical trials. All men underwent a unique rapid biparametric magnetic resonance imaging (IMPROD bpMRI) consisting of T2-weighted imaging and three separate DWI acquisitions. Men with IMPROD bpMRI Likert scores of 3–5 were included. Logistic regression models were developed using IMPROD trial (*n* = 122) and validated using MULTI-IMPROD trial (*n* = 262) data. The model’s performance was evaluated in the terms of PCa detection with Gleason Grade Group 1 (clinically insignificant prostate cancer, iPCa) and > 1 (clinically significant prostate cancer, csPCa). Net benefits and decision curve analyses (DCA) were compared. Combined biopsies were used for reference.

**Results:**

The developed nomogram included age, PSA, prostate volume, MRI suspicion score (IMPROD bpMRI Likert or PIRADsv2.1 score), MRI-suspicion lesion volume percentage, and lesion location. All these variables were significant predictors of csPCa in SBx in multivariable analysis. In the validation cohort (*n* = 262) using different nomogram cutoffs, 19–43% of men would have avoided SBx while missing 1–4% of csPCa and avoiding detection of 9–20% of iPCa. Similar performance was found for nomograms using IMPROD bpMRI Likert score or v2.1.

**Conclusions:**

The developed nomogram demonstrated potential to select men with a clinical suspicion of PCa who would benefit from performing SBx in addition to TBx. Public access to the nomogram is provided at:https://petiv.utu.fi/multiimprod/.

**Electronic supplementary material:**

The online version of this article (10.1007/s00345-020-03393-8) contains supplementary material, which is available to authorized users.

## Introduction

In the recent years, Magnetic Resonance Imaging (MRI) is increasingly used in the diagnostic pathways for prostate cancer (PCa). Simplified MRI protocols with shorter acquisition time and with no use of contrast agents (biparametric MRI) hold the promise to further reduce costs of prostate imaging and promote its use in any patient at risk of PCa [1, 2]. Furthermore, compared to other tools such as biomarkers, prostate MRI allows to characterize and localize PCa lesions and to eventually perform targeted biopsy of any suspicious lesions. Given the reported superiority of the MRI-targeted biopsy (TBx) compared to systematic biopsy (SBx) in recent prospective clinical trials (PROMIS [3], PRECISION [4], and MRI-FIRST [5]), there is a growing interest to evaluate if SBx is needed in addition to TBx in men with a clinical suspicion of PCa. Avoiding SBx would reduce the risks of infection, bleeding and pain associated with additional cores sampling [6, 7]. Additionally, the incidental detection of clinically insignificant PCa (iPCa) is more frequent in SBx than in TBx [8]. Finally, the workload for pathologist evaluating biopsy cores would be reduced from reviewing 12–18 cores to 2–6 cores. However, the risk of missing clinically significant PCa (csPCa) must be addressed. A recent Cochrane meta-analysis of the results of studies evaluating MRI and MRI-targeted biopsy suggests that omitting systematic biopsy would miss approximately 16% of csPCa in biopsy-naive patients and 10% in the repeat-biopsy setting.

Several multivariable models to predict negative SBx have been developed to avoid systematic cores at the time of target biopsy [10, 11]. None of these models showed excellent accuracy, and their clinical benefit is questionable. There are at least three reasons behind TBx failure: (1) Misdiagnosis of the lesion by the radiologist either due to the low quality of prostate MRI or misinterpretation; (2) Presence of csPCa that is not visible on MRI; (3) Targeting error.

Since smaller lesions in big prostates are more likely to be missed by both the reader or by the person performing the TBx, we aimed to develop and externally validate a nomogram based on MRI volumetric parameters and clinical information for deciding when SBx should be performed in addition to TBx in man with suspicious prostate MRI.

## Materials and methods

### Study design and population

We retrospectively analyzed data from two prospective clinical trials involving a total of 499 patients (Supporting Material Figure S1). All patients underwent biparametric MRI according to IMPROD protocol (IMPROD bpMRI). Patients with negative MRI (IMPROD bpMRI Likert score/PIRADsv2.1 score of 1–2) were excluded from the analysis (*n* = 112). Additionally, four men were excluded: three having a PIRADsv2.1 score > 2 while IMPROD bpMRI was Likert 2 and no TBx was performed; one having PIRADsv2.1 score of 1 while IMPROD bpMRI was Likert 3 (Supporting Material Figure S1). Imaging findings for these four men are presented in the Supporting Material (Figure S2–S4) and the following 15 tweet series on twitter https://twitter.com/jambor_ivan/status/1185272940671180800. The final population consisted of two cohort: a single institution development cohort, IMPROD trial (NCT01864135) *n* = 122 and a multi-institution validation cohort, MULTI-IMPROD trial (NCT02241122) *n* = 262. All the study biopsies were taken during 4/2013—05/2017. The patient study flow is shown in Figure S1.

### IMPROD bpMRI protocol and MRI reporting

As described before, the MRI examination was performed using surface array body coils at 3 T in Turku (Verio, Siemens), Helsinki (Skyra, Siemens), and Tampere (Skyra, Siemens), while 1.5 T (Aera, Siemens) MRI scanner was used in Pori [12]. All imaging data sets were prospectively reported by a local radiologist (at least one year of prostate MRI experience at the beginning of the MULTI-IMPROD trial) and confirmed or re-reported centrally by one designated central reader (IJ, 5 years of prostate MRI experience at the beginning of the MULTI-IMPROD trial) to guarantee reporting integrity using a dedicated IMPROD bpMRI reporting system developed before initialization of the trial (See details at https://mrc.utu.fi/mri/improd). Following the completion of the trial, Prostate Imaging Reporting and Data System version 2.1 (PI-RADSv2.1) scores [12] were assigned by the central reader (IJ). Volumetric analysis was performed using manually delineating whole prostate volumes and all MRI suspicious lesions on axial T2-weigthed images.

Volumes were calculated as the sum of prostate/lesion area on each slice multiplied by slice thicknesses (3 mm). Cancer volume on MRI was calculated as the sum of all the lesion volumes in each patient. Total percentage of cancer on MRI was calculated as the ratio of cancer volume on MRI divided the whole prostate volume. The MRI protocols, all MRI data and MRI reports are freely available at the following address: https://petiv.utu.fi/improd/ and https://petiv.utu.fi/multiimprod/.

### Biopsy procedure and histopathological analysis

In IMPROD trial, only the dominant lesion was targeted (two targeted biopsy cores), whereas in MULTI-IMPROD trial, up to two lesions were targeted (two targeted biopsy cores per each lesion). A cognitive targeting method was used with the exception of one center (Helsinki) where a MRI-TRUS fusion platform was used (*n* = 45). Every patient in the present study underwent the first TBx of every MRI suspicious lesion. The same surgeon subsequently performed 12 cores SBx using a sextant template. The location of each SBx core was random and thus may overlap in some degree with MRI lesions. Histopathology of all biopsies was reported separately at each center by a uropathologist, each with at least 5 years of experience in genitourinary pathology at the beginning of the trial, using the 2014 International Society of Urological Pathology modified Gleason grading system [13]. The Gleason score for each patient was assigned using the overall Gleason score from all SBx and TBx cores. Findings from MRI and clinical data were not available for the pathologists.

### Outcome measurements and statistical analysis

Outcomes of this study were the rates of csPCa, Gleason Grade Group (GGG) > 1 and iPCa, GGG = 1, in SBx, TBx and overall (SBx + TBx).

Descriptive characteristics of the development and validation cohorts were calculated. Continuous variables are reported as medians ± standard deviation (when appropriate) and compared by the Mann Whitney test for independent groups. Differences in rates were tested by the chi-square test.

To graphically assess how prostate and lesion dimensions impact the diagnostic accuracy of TBx and SBx, we plotted the actual probability of diagnosing csPCa and iPCa in SBx and TBx according to the percentage of prostate involved by tumor.

Univariable and Multivariable analyses were performed in the development cohort to evaluate predictors of csPCa in SBx, TBx and overall (SBx + TBx).

Two different logistic regression models were developed for each of the three outcomes (csPCa in SBx, TBx, SBx + TBx) including either PI-RADSv2.1 or IMPROD bpMRI Likert score in addition to the other variables. The models with the best area under the curve (AUC) in the development cohort were selected and the linear prediction of the logistic function was used to perform the decision curve analysis in the validation cohort.

Finally, to provide physicians and patients with an outline for deciding when to perform SBx in addition to TBx, we performed the systematic analysis of model-derived cutoffs, considering the predicted probability of the models in the validation cohort. Statistical analyses were performed using Stata 14 (StataCorp LP, College Station, TX, USA). All tests were 2-sided with a significance level set at *p* < 0.05.

## Results

Patients’ characteristic for the included 122 and 262 men in the developmental (IMPROD trial) and validation (MULTI-IMPROD) cohorts, respectively, are presented in Table 1. PSA density (0.20 vs 0.16, *p* 0.004), index lesion volume (0.98 vs 0.73, *p* 0.015) and csPCa detection rate in TBx (60% vs 47%, *p* 0.01) were slightly higher in the developmental cohort but no difference were found in overall csPCa detection rate ( csPCa detection rate in TBx + SBx, *p* 0.051). CsPCa detection rate according to Likert 1–2, 3 and 4–5 lesion were respectively 4.5%, 47.6% and 79.7% in the development cohort, 10.8%, 30.5% and 70.3% in the validation cohort. No difference was found in csPCa detection rate according to PIRADsv2.1 score.

**Table 1:** Descriptive characteristics of the development and validation cohort

	Development cohort IMPROD (*N* = 122)	Validation cohort MULTI IMPROD (*N* = 262)	*p* value
Age (year)	65.5 (59.0, 69.0)	66.0 (59.0, 70.0)	0.6
DRE, *n* (%)			
Negative	93 (76.2%)	182 (69.5%)	0.2
Positive	29 (23.8%)	80 (30.5%)	
PSA, ng/ml	7.5 (5.7, 9.8)	7.2 (5.4, 9.1)	0.10
Prostate volume, ml	39.0 (29.9, 51.5)	41.7 (33.0, 54.3)	0.12
PSA density	0.20 (0.14, 0.27)	0.16 (0.12, 0.23)	0.004
TRUS, *n* (%)			
Negative	78 (63.9%)	180 (68.7%)	0.4
Suspicious	44 (36.1%)	82 (31.3%)	
Cancer volume on MRI, ml	1.08 (0.66, 1.93)	0.97 (0.52, 1.95)	0.3
Total % Cancer volume on MRI, ml	2.78 (1.58, 5.39)	2.39 (1.16, 5.02)	0.12
Index lesion Volume, ml	0.98 (0.61, 1.71)	0.73 (0.41, 1.75)	0.012
PIRADSv2.1, *n* (%)			
3	19 (15.6%)	61 (23.3%)	0.2
4	49 (40.2%)	89 (34.0%)	
5	54 (44.3%)	112 (42.7%)	
IMPROD bpMRI LIKERT, *n* (%)			
3	22 (18.0%)	65 (24.8%)	0.084
4	21 (17.2%)	59 (22.5%)	
5	79 (64.8%)	138 (52.7%)	
Lesion Location, *n* (%)			
PZ	87 (71.3%)	207 (79.0%)	0.10
TZ-CZ	35 (28.7%)	55 (21.0%)	
csPCa overall, *n* (%)	79 (64.8%)	142 (54.2%)	0.051
csPCa on SBx, *n* (%)	60 (49.2%)	129 (49.2%)	1
csPCa on TBx, *n* (%)	74 (60.7%)	122 (46.6%)	0.01

*DRE* digital rectal examination, *PSA* prostate specific antigen, *PZ* peripheral zone, *TZ-CZ* transitional -central zone, *csPCa* clinically significant prostate cancer (Gleason score > 3 + 3), *SBx* systematic biopsy, *TBx* targeted biopsy

### Univariate analysis in the developmental cohort

The results of univariate analysis evaluating predictors of csPCa in SBx, TBx and all cores (SBx + TBx) are shown in Table 2. In contrast to TBx, MRI suspicious lesion location and prostate volume were significant predictors of csPCa in SBx with *p* values of 0.039 and 0.02, respectively. The remaining significant predictors of csPCa in SBx were similar to TBx. The total volume of the suspicious lesion on MRI, index lesion volume and the percentage of prostate involved by cancer emerged as important predictors csPCa in SBx, TBx and all cores (SBx + TBx).

**Table 2:** Univariable analysis evaluating predictors of csPCa in systematic, target, and all cores in the development cohort

Covariate	csPCa on SBx			csPCa on TBx			csPCa overall		
	OR	95% CI	*p*	OR	95% CI	*p*	OR	95% CI	*p*
Age, per year	1.05	0.99, 1.11	0.102	1.08	1.01, 1.14	**0.017**	1.06	1.00, 1.12	0.060
Dre									
Negative	Ref			Ref			Ref		
Suspicious	53.37	6.94, 410.49	**< 0.001**	8.12	2.30, 28.71	**0.001**	23.06	3.01, 176.63	**0.003**
Psa, ng/ml	1.10	1.00, 1.21	**0.051**	1.14	1.02, 1.27	**0.024**	1.12	1.00, 1.25	**0.048**
Prostate volume	0.98	0.95, 1.00	**0.020**	0.99	0.97, 1.01	0.309	0.98	0.96, 1.00	0.100
PSA density, per 0.1	1.59	1.15, 2.21	**0.005**	1.47	1.05, 2.07	**0.025**	1.62	1.11, 2.38	**0.013**
TRUS									
Negative	Ref			Ref			Ref		
Suspicious	8.24	3.44, 19.74	**< 0.001**	5.86	2.33, 14.73	**< 0.001**	5.72	2.17, 15.06	**< 0.001**
Cancer volume on MRI, ml	1.84	1.28, 2.66	**0.001**	1.65	1.14, 2.39	**0.008**	2.38	1.37, 4.14	**0.002**
Total % Cancer volume on MRI	1.35	1.15, 1.60	**< 0.001**	1.18	1.03, 1.34	**0.015**	1.54	1.22, 1.95	**< 0.001**
Index lesion Volume, ml	1.75	1.21, 2.53	**0.003**	1.60	1.09, 2.36	**0.016**	2.33	1.32, 4.12	**0.004**
PIRADSv.2.1									
3*	Ref			Ref			Ref		
4	4.94	1.02,23.87	**0.047**	7.11	1.83, 27.62	**0.005**	7.73	1.99, 30.08	**0.003**
5	24.29	4.97,118.68	**< 0.001**	20.85	5.14, 84.52	**< 0.001**	35.81	8.26, 155.22	**< 0.001**
IMPROD bpMRI LIKERT									
3*	Ref			Ref			Ref		
4	8.40	0.91,77.21	0.060	19.09	2.16, 169.09	**0.008**	19.09	2.16, 169.09	**0.008**
5	42.81	5.45,335.95	** < 0.001**	82.69	10.33, 661.72	**< 0.001**	129.82	15.82, 1065.2	**< 0.001**
Lesion location									
PZ	Ref			Ref			Ref		
TZ-CZ	0.42	0.19,0.96	**0.039**	0.69	0.31, 1.53	0.362	0.89	0.39, 2.01	0.781

*PZ* peripheral zone, *TZ-CZ* transitional-central zone, *csPCa* clinically significant prostate cancer (Gleason score > 3 + 3), *SBx* systematic biopsy, *TBx* targeted biopsy

*p values are presented with respect to variables with “Ref”. All values with *p* value < 0.05 are in bold

**Fig. 1:**
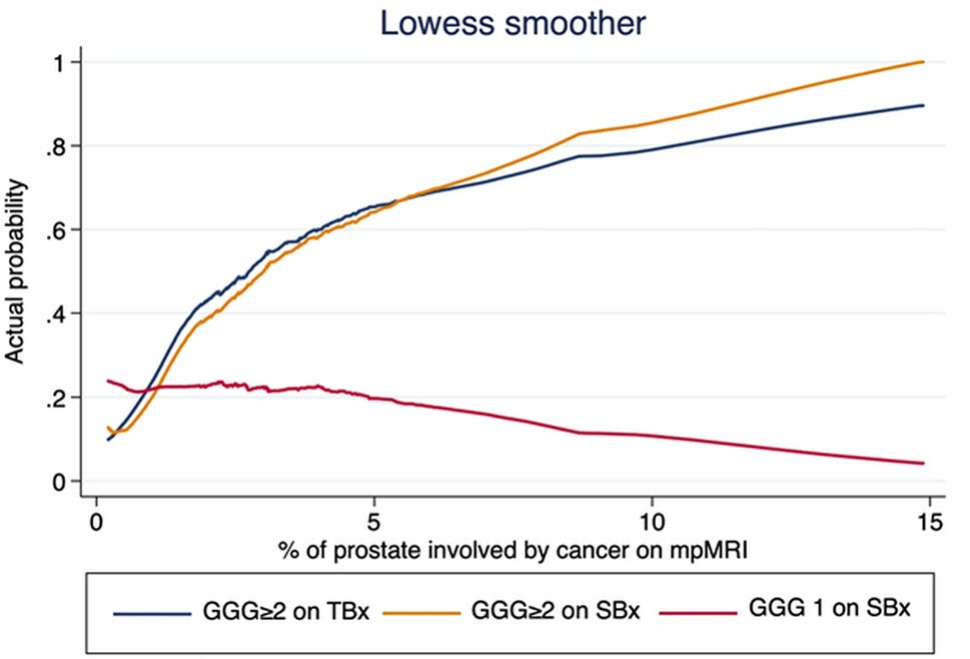
Actual Probability of Prostate cancer diagnosis in systematic and targeted biopsy cores as a function of MRI-suspicion lesion volume percentage

Figure 1 graphically shows that the actual probability of diagnosing csPCa in SBx and TBx increase with the increase of the percentage of prostate involved by a suspicious lesion on MRI. However, in patients with low percentages, the probability of finding csPCa in SBx only is very low and actually lower than the probability of finding iPCa.

### Multivariable analysis in the developmental cohort

The results of multivariable analysis evaluating predictors of csPCa in SBx using IMPROD bpMRI Likert score and PI-RADsv2.1 are shown in Table 3 and Supporting Material Table S1, respectively. The corresponding results for predictors of csPCa in TBx are shown in Table 4 and Supporting Material Table S2, respectively. Finally, the results of multivariable analysis evaluating predictors of csPCa in SBx + TBx using IMPROD bpMRI Likert score and PI-RADsv2.1 are shown in Supporting Material Table S3 and S4, respectively. In contrast to predictors of csPCa in TBx and SBx + TBx, PSA and MRI-suspicion lesion location were a significant predictor of csPCa only in SBx (Table 3 and Supporting Material Table S1). Decision curve analysis for SBx, TBx, and SBx + TBx are shown in Supporting Material Figure S6, S7, and S8, respectively.

**Table 3:** Multivariable analysis to predict clinically significant prostate cancer (Gleason score > 3 + 3) in systematic cores using IMPROD bpMRI Likert scoring system

Covariate	Development cohort IMPROD (*N* = 122) AUC:0.8594			Validation cohort MULTI IMPROD (*N* = 262) AUC: 0.8728		
	OR	95% CI	*p*	OR	95% CI	*p*
Age, per year	1.11	1.02, 1.20	0.011	1.06	1.02, 1.11	0.006
Psa, ng/ml	1.07	0.93, 1.24	0.349	1.11	1.00, 1.23	0.060
Prostate volume	0.97	0.95, 1.00	0.082	0.98	0.96, 1.00	0.036
IMPROD bpMRI LIKERT						
3*	1			1		
4	10.38	1.02, 105.21	0.048	4.10	1.46, 11.53	0.007
5	24.58	2.82, 214.50	0.004	12.93	4.77, 35.03	< 0.001
Total % Cancer volume on MRI	1.24	1.03, 1.50	0.025	1.12	1.02, 1.24	0.018
Lesion Location						
PZ	1			1		
TZ-CZ	0.19	0.06, 0.62	0.006	0.30	0.13, 0.68	0.004

*p values are presented with respect to variables with “Ref”

**Table 4:** Multivariable analysis to predict clinically significant prostate cancer (Gleason score > 3 + 3) in target biopsy cores using IMPROD bpMRI Likert scoring system

Covariate	Development cohort IMPROD (*N* = 122) AUC: 0.8649			Validation cohort MULTI IMPROD (*N* = 262) AUC: 0.8320		
	OR	95% CI	*p*	OR	95% CI	*p*
Age, per year	1.11	1.03, 1.20	0.007	1.06	1.02, 1.10	0.005
Psa density, per 0.1	1.06	0.73, 1.55	0.765	1.35	0.98, 1.88	0.070
IMPROD bpMRI LIKERT						
3*	Ref			Ref		
4	23.59	2.52, 221.07	0.006	3.30	1.24, 8.77	0.017
5	90.87	10.20, 809.16	< 0.001	11.35	4.54, 28.35	< 0.001
Total Cancer volume on MRI, ml	1.20	0.79, 1.82	0.396	1.14	0.98, 1.33	0.091

*p values are presented with respect to variables with “Ref”

### Risk calculator

A risk calculator for deciding when SBx should be performed in addition to TBx in men with suspicious MRI findings was developed using the developmental cohort (IMPROD, *n* = 122) while the results using the validation cohort (MULTI-IMPROD, *n* = 262) are shown in Table 5. The developed nomogram included age, PSA, prostate volume, MRI suspicion score (IMPROD bpMRI Likert or PIRADsv2.1 score), MRI-suspicion lesion volume percentage, and lesion location. Beta coefficients of the logit function are shown in Supporting Material Table S5-S6.

**Table 5:** Systematic analysis of the nomogram-derived cutoffs to help identify patients who are going to benefit from systematic cores in addition to target cores in the validation cohort

Nomogram calculated probability, cutoff (%)	Patients resulting below cutoff, *n* (%)^a^	iPCa on SBx, *n* (%)^b^	csPCa on SBx, *n* (%)^c^	Clinical Implication	
				csPCa missed, *n* (%)^d^	iPCa not detected, *n* (%)^e^
PIRADSv 2.1 Model					
5	26 (9.9)	24 (18)	2 (1.6)	2 (1.4)	2 (3.7)
7.5	41 (15.6)	36 (27.1)	5 (3.9)	3 (2.1)	3 (5.6)
10	58 (22.1)	51 (38.3)	7 (5.4)	4 (2.8)	6 (11.1)
12.5	63 (24)	55 (41.4)	8 (6.2)	4 (2.8)	7 (13)
15	69 (26.3)	60 (45.1)	9 (7)	4 (2.8)	8 (14.8)
17.5	75 (28.6)	65 (48.9)	10 (7.8)	4 (2.8)	9 (16.7)
20	86 (32.8)	73 (54.9)	13 (10.1)	4 (2.8)	10 (18.5)
22.5	95 (36.2)	78 (58.6)	17 (13.2)	6 (4.2)	10 (18.5)
25	101 (38.5)	82 (61.7)	19 (14.7)	6 (4.2)	11 (20.4)
27.5	105 (40.1)	85 (63.9)	20 (15.5)	6 (4.2)	12 (22.2)
30	112 (42.7)	92 (69.2)	20 (15.5)	6 (4.2)	12 (22.2)
IMPROD bpMRI LIKERT Model					
5	50 (19.1)	47 (35.3)	3 (2.3)	2 (1.4)	5 (9.3)
7.5	61 (23.3)	55 (41.4)	6 (4.7)	3 (2.1)	6 (11.1)
10	75 (28.6)	68 (51.1)	7 (5.4)	3 (2.1)	7 (13)
12.5	80 (30.5)	70 (52.6)	10 (7.8)	4 (2.8)	8 (14.8)
15	87 (33.2)	75 (56.4)	12 (9.3)	5 (3.5)	8 (14.8)
17.5	91 (34.7)	76 (57.1)	15 (11.6)	5 (3.5)	8 (14.8)
20	94 (35.9)	79 (59.4)	15 (11.6)	5 (3.5)	8 (14.8)
22.5	100 (38.2)	82 (61.7)	18 (14)	5 (3.5)	8 (14.8)
25	102 (38.9)	84 (63.2)	18 (1)	5 (3.5)	9 (16.7)
27.5	105 (40.1)	87 (65.4)	18 (14)	5 (3.5)	9 (16.7)
30	111 (42.4)	92 (69.2)	19 (14.7)	6 (4.2)	11 (20.4)

The table shows the patients resulting below the cut-off and the clinical implication of avoiding SBx in these men

^a^Out of the total number of biopsies performed (*n* = 262)

^b^Out of the total number of iPCa in SBx (*n* = 133);

^c^Out of the total number of csPCa in SBx (*n* = 129); ^d^ out of the total number of csPCa in any core (*n* = 142);

^e^Out of the total number of iPCa diagnosed (*n* = 54)

The external validity of the risk calculator was performed in the validation cohort (*n* = 262) using DCA and evaluating the clinical utility of the risk calculator at different cut-offs (Table 5). DCA showed a net clinical benefit for every value of predicted probability above 10%.

The systematic analysis of the model derived cutoffs revealed that 19–43% of men would have avoided SBx while missing 1–4% of csPCa and avoiding detection of 9–20% of iPCa (Table 5). Similar performance was found for nomograms using IMPROD bpMRI Likert score or PIRADsv2.1.

## Discussion

In this study, using data of two prospective trials, we developed a novel instrument based onto readily-available clinical parameters (age, PSA, prostate volume, IMPROD bpMRI Likert or PIRADsv2.1 score, total percentage of cancer volume on MRI and lesion location) that can help the physician to understand the added value of SBx in patients who underwent TBx of MRI suspicious lesion.

Avoiding SBx in patients who have a suspicious MRI lesion represents a great challenge for a urologist.

In an ideal world, where MRI and targeting systems show perfect performance, avoiding SBx would result in less complications and a lower rate of diagnosis of iPCa.

Recently Wegelin et al., in a post hoc analysis of a randomized controlled trial, compared adverse events among three mpMRI-based TBx techniques of the prostate in men with prior negative SBx and a persisting suspicion of PCa [6, 14]. No difference were found in PCa detection rates between in-bore MRI TBx, MRI-TRUS fusion TBx, and cognitive registration TRUS TBx [14], however, the number of biopsy cores taken was different between the various techniques and was significantly associated with the occurrence of adverse events (OR 1.11 [95% CI 1.06–1.17, *p* < 0.001]) [6].

To the best of our knowledge no level 1 evidence is available in the biopsy naïve setting. Indeed, when mpMRI is positive (i.e. PIRADsv2.1 > 3), EAU guidelines suggest performing 12-cores SBx in addition to TBx of every suspicious lesion in biopsy naïve patients (Level of evidence: 2A; strength rating: strong) and TBx only in patients with prior negative biopsy (Level of evidence: 2A; strength rating: weak) [15].

Although this might not be feasible in every center at the moment, the aim of this study was to find a subset of biopsy naïve men with a clinical suspicion of PCa that can safely avoid 12-core SBx without compromising the detection of csPCa.

Unlike prior studies, we considered MRI volumetric parameters and we found that adding the prostate volume and percentage of prostate involved by tumor to a model based on age, PSA, lesion location and lesion suspicious score, increased the model predictive accuracy for csPCa on SBx from 0.82 to 0.86 for the model using IMPROD bpMRI Likert and from 0.80 to 0.85 for the model using PIRADsv2.1. Our rationale for this decision was that smaller lesions in big prostates are more likely to be missed by a reader interpreted prostate MRI or by the person performing TBx.

Turning findings into clinical practice, in a 56 years old man with PSA 4 ng/ml and one PIRADsv2.1 4 lesion of 1 cm (0.52 mL), the estimated probability of finding csPCa in SBx is 10.4% in case of prostate volume 60 mL (0.87% of prostate involved by tumor), as opposed to 5.7% in case of prostate volume 80 mL (0.65% of prostate involved by tumor).

Among the models for prediction of sPC in SBx, the nomogram developed by Sathianathen et al. considered the clinical setting (biopsy naive, previous negative biopsy and Active surveillance patients), number of MRI lesions and the highest PIRADS score [11]. Such model showed clinical benefit in a range of probability between 10 and 30%. The lack of external validation limits the generalization of these findings. Moreover, the model was developed to predict csPCa in systematic cores only (with negative TBx). Indeed, our outcome was intentionally csPCa in SBx irrespective of the results of TBx. We believe that information on every positive core (from TBx or SBx) are still necessary when planning focal therapies and to assess the oncologic outcome of the patient since the current risk stratification tools take into account the number of positive cores (e.g. NCCN risk categories that stratifies patients into favorable or unfavorable intermediate-risk based on the number of cores with GGG 2 [16]).

Similar to our risk calculator, the model developed by Stabile et al. predicts csPCa in SBx. Their model ultimately included age, PSA, Prostate volume, number of MRI lesions, PIRADS score and the biopsy setting (biopsy naive vs previous negative biopsy patients) [10]. However, the DCA showed modest clinical benefit in the external validation cohort and the authors concluded that the number of systematic biopsies spared compared with the number of aggressive PCa missed is negligible. Moreover, no useful clinical model can be developed to safely identify those patients who could avoid SBx in addition to TBx. Conversely, our risk calculator showed a clinical benefit in the external validation cohort for the low threshold of probabilities. The addition of volumetric parameters derived from MRI seems to be the key to develop nomograms that help in this scenario.

Biomarkers such us a 4Kscore, selectMDx or the Stockholm3 test could also help to predict the risk of csPCa in SBx in patients with a positive MRI. However, the evidence in this regard is very limited and while MRI is becoming widely available, molecular tests are not yet used in routine clinical practice [17–20].

The present study is not devoid of limitations. First, we acknowledge that we used a cohort of patients enrolled in a prospective clinical trial that received MRI and biopsies according to the highest standard of care with a central prospective review of MRI images for prostate cancer detection. Although, this is a reflective of the daily clinical practice in referral academic centers it may be limit the validity of our findings to nonacademic centers where radiologists and urologists are still in the initial phase of their learning curve. Moreover, we used the combination of SBx + TBx rather than the Radical prostatectomy specimen or template transperineal saturation biopsy as a reference standard. Even if the previous studies showed a certain rate of discordance between Bx GGG and final pathology GGG [21–23], this still represents the standard of care and none of the two other options would have been feasible.

## Conclusions

In conclusion, patients with a small lesion on MRI in big prostate benefit less from standard cores at the time of MRI-targeted biopsy. We developed and externally validated in a multi-institutional cohort a risk calculator to predict the added values of SBx to TBx that could help urologist avoid unnecessary biopsy sampling, reduce detection of iPCa while maintaining detection of csPCa.

## Electronic supplementary material

Below is the link to the electronic supplementary material.

Supplementary file1 (DOCX 1115 kb)

## Data Availability

Free public access to IMPROD bpMRI protocol, all IMPROD and MULTI-IMPROD data sets, MRI reports, clinical data with pathology and follow up data is provided on the trials’ servers: https://petiv.utu.fi/improd/ and https://petiv.utu.fi/multiimprod/. Free public access to the developed model is provided at https://petiv.utu.fi/multiimprod/.

## Abbreviations

csPCa: Clinically significant PCa
iPCa: Clinically insignificant PCa iPCa
SBx: Magnetic Resonance Imaging-targeted biopsy
TBx: Systematic biopsy
MRI: Magnetic Resonance Imaging
