# Supplementary material for: Added value of systematic biopsy in men with a clinical suspicion of prostate cancer undergoing biparametric MRI-targeted biopsy: multi-institutional external validation study
Source: World J Urol. 2020 Aug 10;39(6):1879–87. doi: 10.1007/s00345-020-03393-8 (PMC8217016; doi:10.1007/s00345-020-03393-8)

**Added value of systematic biopsy in men with a clinical suspicion of prostate cancer undergoing MRI-targeted biopsy: Multi-institutional external validation study**

**Supporting Material**

**Table S1** Multivariate analysis to predict clinically significant prostate cancer (Gleason score >3+3) in systematic biopsy cores using PIRADS scoring system

|  | **Development cohort**  **IMPROD (N=122)**  **AUC: 0.8551** | | | **Validation cohort**  **MULTI IMPROD (N=262)**  **AUC: 0.8645** | | |
| --- | --- | --- | --- | --- | --- | --- |
| **Covariate** | **OR** | **95% C.I.** | **P>\|z\|** | **OR** | **95% C.I.** | **P>\|z\|** |
| **Age, per y** | 1.10 | 1.02,1.19 | 0.011 | 1.06 | 1.02,1.11 | 0.006 |
| **Psa, ng/ml** | 1.06 | 0.92,1.22 | 0.394 | 1.12 | 1.00,1.24 | 0.041 |
| **Prostate volume** | 0.97 | 0.94,1.00 | 0.041 | 0.98 | 0.96,1.00 | 0.025 |
| **PIRADS** |  |  |  |  |  |  |
| 3* | Ref. |  |  | Ref. |  |  |
| 4 | 4.27 | 0.80,22.76 | 0.089 | 5.08 | 1.91,13.47 | 0.001 |
| 5 | 12.37 | 2.15,71.07 | 0.005 | 11.73 | 4.01,34.32 | <0.001 |
| **Total % Cancer volume on MRI, ml** | 1.22 | 1.01,1.48 | 0.038 | 1.12 | 1.01,1.24 | 0.029 |
| **Lesion Location** |  |  |  |  |  |  |
| PZ | Ref. |  |  | Ref. |  |  |
| TZ-CZ | 0.20 | 0.06,0.63 | 0.006 | 0.26 | 0.11,0.60 | 0.002 |

* - p-values are presented with respect to variables with “Ref”.

**Table S2** Multivariate analysis to predict clinically significant prostate cancer (Gleason score >3+3) in targeted biopsy cores using PI-RADsv2.1 scoring system

|  | **Development cohort**  **IMPROD (N=122)**  **AUC: 0.7976** | | | **Validation cohort**  **MULTI IMPROD (N=262)**  **AUC: 0.8169** | | |
| --- | --- | --- | --- | --- | --- | --- |
| **Covariate** | **OR** | **95% C.I.** | **P>\|z\|** | **OR** | **95% C.I.** | **P>\|z\|** |
| **Age, per y** | 1.09 | 1.02,1.17 | 0.011 | 1.06 | 1.02,1.10 | 0.003 |
| **Psa density, per 0.1** | 1.23 | 0.85,1.79 | 0.277 | 1.42 | 1.03,1.97 | 0.033 |
| **PIRADS** |  |  |  |  |  |  |
| 3* | Ref. |  |  | Ref. |  |  |
| 4 | 6.94 | 1.71,28.09 | 0.007 | 4.34 | 1.74,10.81 | 0.002 |
| 5 | 16.16 | 3.53,73.96 | <0.001 | 9.88 | 3.77,25.91 | <0.001 |
| **Total Cancer volume on MRI, ml** | 1.17 | 0.81,1.68 | 0.408 | 1.14 | 0.97,1.33 | 0.110 |

* - p-values are presented with respect to variables with “Ref”.

**Table S3** Multivariate analysis to predict clinically significant prostate cancer (Gleason score >3+3) in systematic and targeted biopsy cores using IMPROD bpMRI Likert scoring system.

|  | **Development cohort**  **IMPROD (N=122)**  **AUC: 0.9017** | | | **Validation cohort**  **MULTI IMPROD (N=262)**  **AUC: 0.8660** | | |
| --- | --- | --- | --- | --- | --- | --- |
| **Covariate** | **OR** | **95% C.I.** | **P>\|z\|** | **OR** | **95% C.I.** | **P>\|z\|** |
| **Age, per y** | 1.10 | 1.01,1.19 | 0.024 | 1.06 | 1.02,1.11 | 0.004 |
| **PSA density, per 0.1** | 1.02 | 0.67,1.55 | 0.927 | 1.40 | 0.97,2.02 | 0.072 |
| **LIKERT** |  |  |  |  |  |  |
| 3* | Ref. |  |  | Ref. |  |  |
| 4 | 28.96 | 2.86,293.65 | 0.004 | 3.99 | 1.57,10.13 | 0.004 |
| 5 | 136.41 | 14.55,1279.12 | <0.001 | 15.15 | 6.08,37.76 | <0.001 |
| **Cancer volume on MRI, ml** | 1.86 | 0.92,3.76 | 0.086 | 1.30 | 1.01,1.67 | 0.041 |

* - p-values are presented with respect to variables with “Ref”.

**Table S4** Multivariate analysis to predict clinically significant prostate cancer (Gleason score >3+3) in systematic and targeted biopsy cores using PI-RADsv2.1 scoring system.

|  | **Development cohort**  **IMPROD (N=122)**  **AUC: 0.8328** | | | **Validation cohort**  **MULTI IMPROD (N=262)**  **AUC: 0.8534** | | |
| --- | --- | --- | --- | --- | --- | --- |
| **Covariate** | **OR** | **95% C.I.** | **P>\|z\|** | **OR** | **95% C.I.** | **P>\|z\|** |
| **Age, per y** | 1.07 | 1.00,1.15 | 0.045 | 1.06 | 1.02,1.11 | 0.004 |
| **Psa density, per 0.1** | 1.25 | 0.83,1.88 | 0.283 | 1.46 | 1.02,2.10 | 0.038 |
| **PIRADS** |  |  |  |  |  |  |
| 3* | Ref. |  |  | Ref. |  |  |
| 4 | 6.91 | 1.71,27.97 | 0.007 | 4.88 | 2.04,11.70 | <0.001 |
| 5 | 21.77 | 4.48,105.86 | <0.001 | 15.09 | 5.59,40.74 | <0.001 |
| **Cancer volume on MRI, ml** | 1.47 | 0.86,2.51 | 0.157 | 1.25 | 0.97,1.61 | 0.092 |

* - p-values are presented with respect to variables with “Ref”.

**Table S5** Beta coefficients of the logit function to compute the linear prediction of IMPROD bpMRI LIKERT model predicting significant PCa in SBx.

| **Covariate** | **Coef.** | **95% CI** | **p value** |
| --- | --- | --- | --- |
| **Age, per y** | 0.104 | 0.024, 0.184 | 0.011 |
| **Psa, ng/ml** | 0.069 | -0.076, 0.214 | 0.349 |
| **Prostate volume** | -0.026 | -0.055, 0.003 | 0.082 |
| **IMPROD bpMRI LIKERT** |  |  |  |
| 3 | Ref. |  |  |
| 4 | 2.340 | 0.024, 4.656 | 0.048 |
| 5 | 3.202 | 1.035, 5.368 | 0.004 |
| **Total % Cancer volume on MRI** | 0.217 | 0.027, 0.406 | 0.025 |
| **Lesion Location** |  |  |  |
| PZ | Ref. |  |  |
| TZ-CZ | -1.668 | -2.853, -0.482 | 0.006 |
| **Constant** | -9.286 | -14.800, -3.773 | 0.001 |

$$logit\left( \hat{\pi}_{i} \right)=\ln\left( \frac{\hat{\pi}_{i}}{1-\hat{\pi}_{i}} \right)=-9.286+ 0.104(AGE) + 0.069(PSA) - 0.026(Prostate Volume) +2.34(LIKERT3) + 3.202 (LIKERT 5)+0.217(Total \% Cancer volume on MRI) -1.668 (TZ-CZ Lesion Location)$$

**Table S6** Beta coefficients of the logit function to compute the linear prediction of PIRADS model predicting significant PCa in SBx.

| **Covariate** | **Coef.** | **95% CI** | **p value** |
| --- | --- | --- | --- |
| **Age, per y** | 0.099 | 0.022, 0.175 | 0.011 |
| **Psa, ng/ml** | 0.061 | -0.079, 0.201 | 0.394 |
| **Prostate volume** | -0.031 | -0.060, -0.001 | 0.041 |
| **PIRADS** |  |  |  |
| 3 | Ref. |  |  |
| 4 | 1.453 | -0.220, 3.125 | 0.089 |
| 5 | 2.515 | 0.767, 4.264 | 0.005 |
| **Total % Cancer volume on MRI** | 0.201 | 0.011, 0.391 | 0.038 |
| **Lesion Location** |  |  |  |
| PZ | Ref. |  |  |
| TZ-CZ | -1.596 | -2.735, -0.458 | 0.006 |
| **Constant** | -7.704 | -12.689, -2.719 | 0.002 |

$$logit\left( \hat{\pi}_{i} \right)=\ln\left( \frac{\hat{\pi}_{i}}{1-\hat{\pi}_{i}} \right)=-7.704 + 0.099\left( AGE \right)+ 0.061\left( PSA \right)- 0.031\left( Prostate Volume \right)+ 1.453\left( PIRADS 4 \right)+ 2.515\left( PIRADS 5 \right) + 0.201(Total \% Cancer volume on MRI$$

**Figure S1** Study flow

**
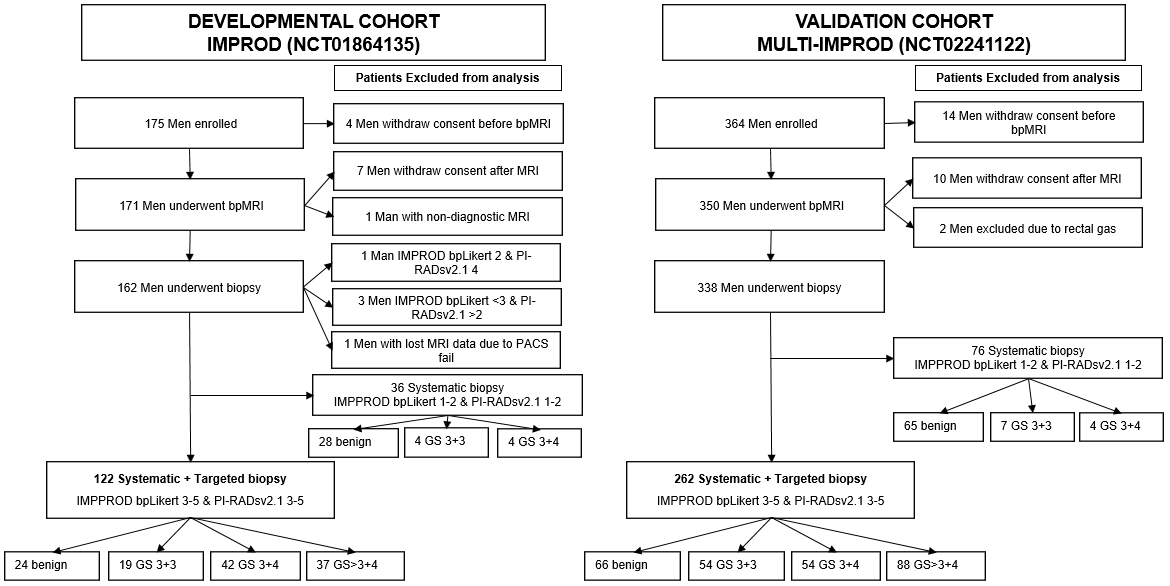
**

**Figure S2** IMPROD trial cases no. 25: IMPROD bpMRI Likert score of 2 and PI-RADSv2.1 of 4. Targeted biopsy was NOT performed. All 12 biopsy cores of systematic biopsy. Stable PSA for over 2 years. A= Axial T2-weighted imaging, B= ADCm of DWI collected using b values of 0-500 s/mm2, C= trace DWI b=1500 s/mm2, D= trace DWI b=2000 s/mm2. Details at: <http://petiv.utu.fi/improd/>


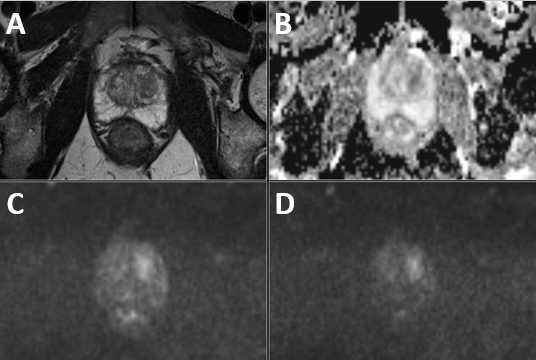


**Figure S3** IMPROD trial cases no. 152: IMPROD bpMRI Likert score of 2 and PI-RADSv2.1 of 3. Targeted biopsy was NOT performed. All 12 biopsy cores of systematic biopsy. Stable PSA for over 1 year. A= Axial T2-weighted imaging, B= ADCm of DWI collected using b values of 0-500 s/mm2, C= trace DWI b=1500 s/mm2, D= trace DWI b=2000 s/mm2. Details at: <http://petiv.utu.fi/improd/>

**
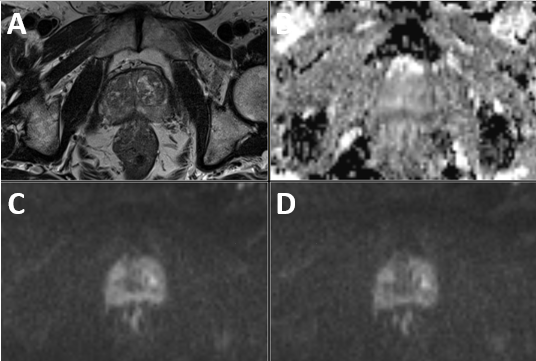
**

**Figure S4** IMPROD trial cases no. 162: IMPROD bpMRI Likert score of 2 and PI-RADSv2.1 of 3. Targeted biopsy was NOT performed. All 12 biopsy cores of systematic biopsy. Stable PSA for over 1 year. A= Axial T2-weighted imaging, B= ADCm of DWI collected using b values of 0-500 s/mm2, C= trace DWI b=1500 s/mm2, D= trace DWI b=2000 s/mm2. Details at: <http://petiv.utu.fi/improd/>

**
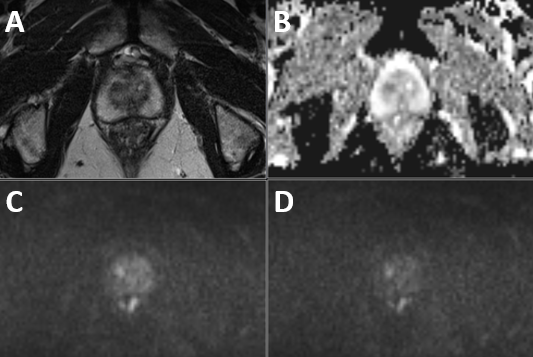
**

**Figure S5** IMPROD trial cases no. 94: IMPROD bpMRI Likert score of 3 and PI-RADSv2.1 of 2. Targeted biopsy was performed to the lesion anterior to the urethra, located in the central gland, mid-gland, right side. Both targeted biopsy cores were benign as well as all 12 biopsy cores of systematic biopsy. Follow up MRI performed 351 days after initial MRI was read as IMPROD bpMRI Likert score of 2, PSA was stable. A= Axial T2-weighted imaging, B= ADCm of DWI collected using b values of 0-500 s/mm^2^, C= trace DWI b=1500 s/mm^2^, D= trace DWI b=2000 s/mm^2^. Details at: <http://petiv.utu.fi/improd/>


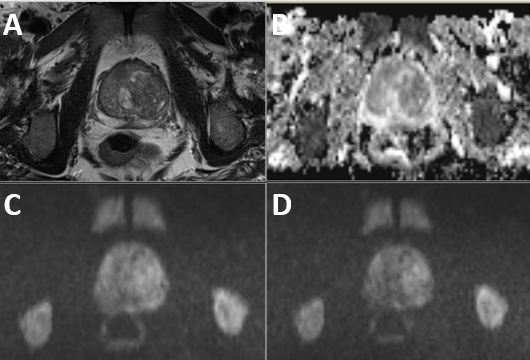


**Figure S6** Decision curve analysis (DCA) showing the net benefit of two models (PIRADS score and IMPROD bpMRI Likert score) for the detection of clinically significant prostate cancer in standard biopsy cores. The DCA simulates two scenarios: one in which all patients would receive SBx+TBx (all) and one in which none undergoes SBx but only TBx (none). The graph gives the expected net benefit per patient when you perform SBx in addition to TBx using different cut-offs of the model derived probability.The models are clinically useful in the range of predicted probabilities where the line lays above the simulated scenarios (above 10%).


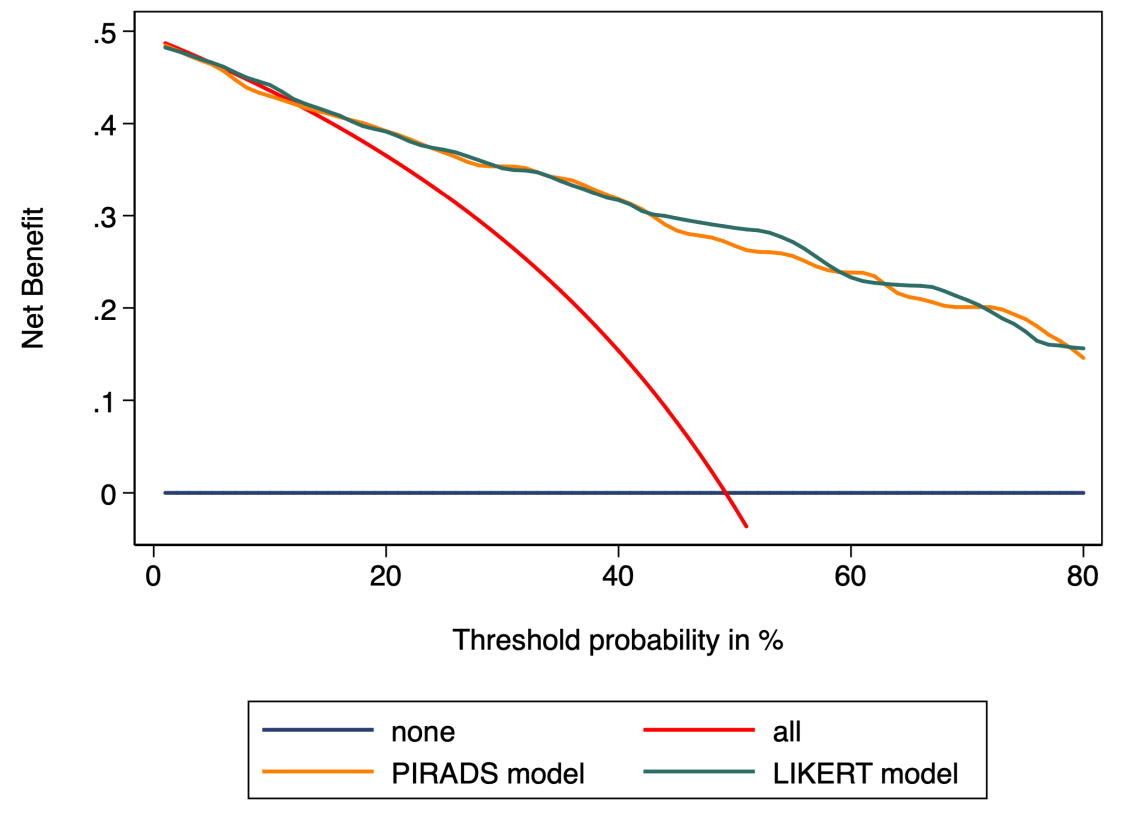


**Figure S7** Decision curve analysis (DCA) showing the net benefit of two model (PIRADS score and IMPROD bpMRI Likert score) for the detection of clinically significant prostate cancer in target biopsy cores. The DCA simulates two scenarios: one in which all patients would receive SBx+TBx (all) and one in which none undergoes TBx (none). The graph gives the expected net benefit per patient when you perform TBx in addition to SBx using different cut-offs of the model derived probability. The models are clinically useful in the range of predicted probabilities where the line lays above the simulated scenarios.


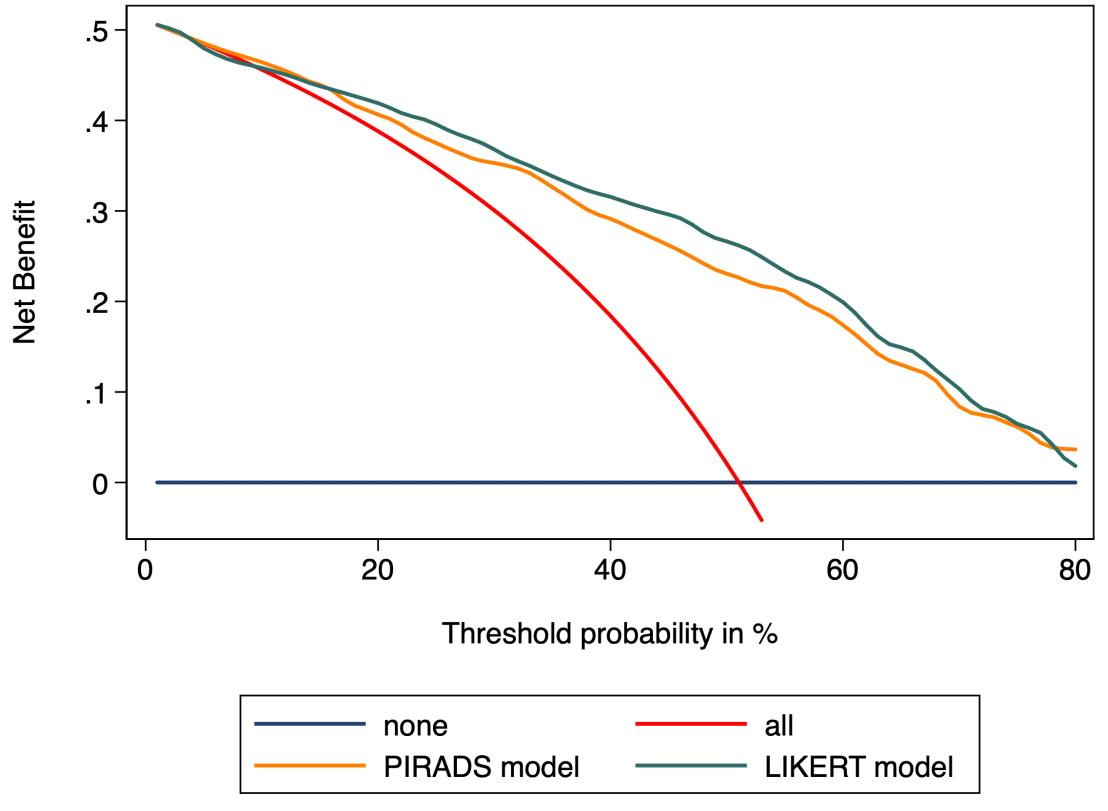


**Figure S8** Decision curve analysis (DCA) showing the net benefit of two model (PIRADS score and IMPROD bpMRI Likert score) for the detection of clinically significant prostate cancer in Any core (SBx+TBx). The DCA simulates two scenarios: one in which all patients would receive SBx+TBx (all) and one in which none undergoes biopsy (none). The models are clinically useful in the range of predicted probabilities where the line lays above the simulated scenarios.


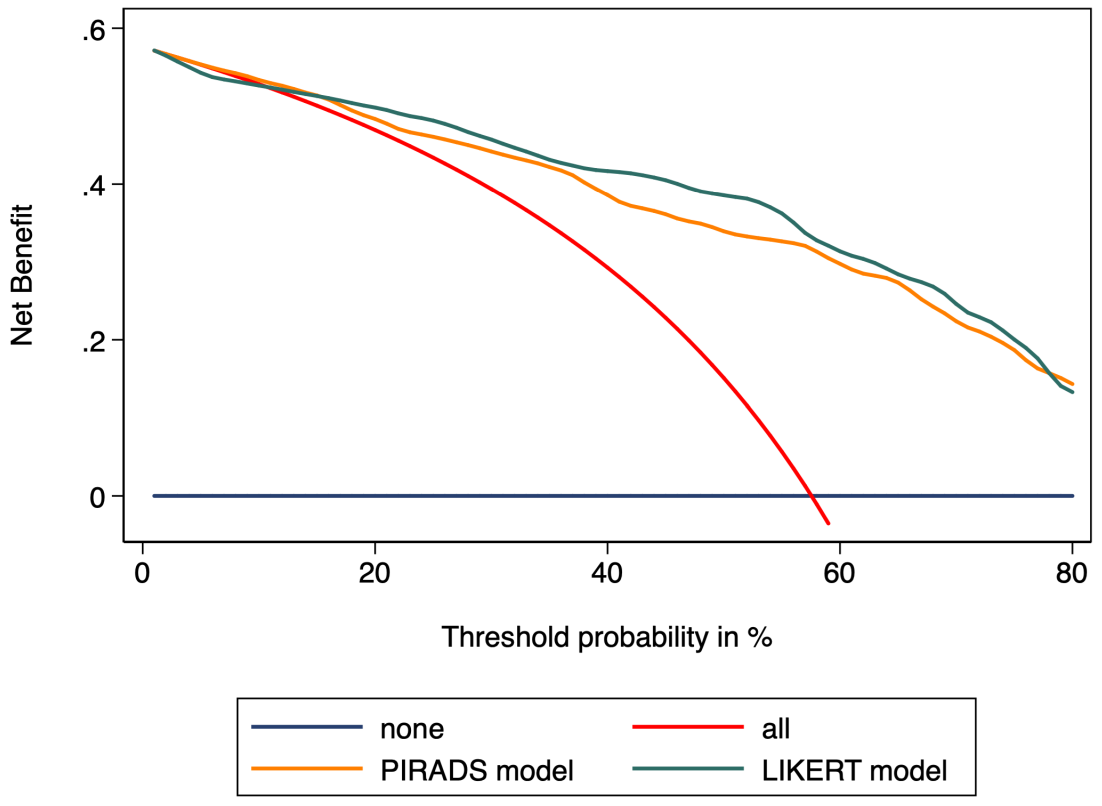

Supplement: Supplementary file 1 — Supplementary file1 (DOCX 1115 kb) [file 345_2020_3393_MOESM1_ESM.docx]
